# Tissue content of mucins in the colonic mucosa with and without fecal stream: a clinical study

**DOI:** 10.1590/0102-672020260000047e1976

**Published:** 2026-10-05

**Authors:** Bruna Zini de PAULA FREITAS, José Aires PEREIRA, Fabio Guilherme CAMPOS, Geovanna Pacciulli PEREIRA, Poliana Pacciulli PEREIRA, Luísa Motta JUSTO, Carlos Augusto Real MARTINEZ

**Affiliations:** 1 Universidade Estadual de Campinas – Campinas (SP), Brazil.; 2 Universidade São Francisco, Faculty of Medicine – Bragança Paulista (SP), Brazil.; 3 Universidade de São Paulo, Faculty of Medicine – São Paulo (SP), Brazil.; 4 Pontifícia Universidade Católica de São Paulo, Faculty of Medical Sciences – Sorocaba (SP), Brazil.

**Keywords:** Proctitis, Fatty Acids. Volatile, Mucins, Proctite, Ácidos Graxos Voláteis, Mucinas

## Abstract

**Background:**

Alterations in mucin expression in the intestinal mucosa are among the main histological findings in inflammatory colonic diseases. No previous study has quantified the tissue expression of neutral and acidic mucins, including sulfomucins and sialomucins, in patients with diversion colitis.

**Aims:**

To quantify the tissue content of neutral and acidic mucins (sulfomucins and sialomucins) in colonic mucosa with and without intestinal transit.

**Methods:**

Forty patients undergoing elective colonoscopy at the Hospital de Clínicas – Unicamp were included. Group 1 comprised 20 patients with an ileostomy or colostomy for more than two months. Group 2 (control) included 20 patients without any type of ostomy. Patients with inflammatory bowel disease or previous pelvic radiotherapy were excluded. Three mucosal biopsies were obtained from each patient during colonoscopy. Neutral and acidic mucins were assessed using Periodic Acid-Schiff and Alcian Blue staining, respectively. Acidic mucin subtypes (sulfomucins and sialomucins) were identified using Alcian Blue-high iron diamine. Tissue content was quantified by computer-assisted image analysis. Statistical analysis compared mucin levels between segments with and without fecal transit, with significance set at p<0.05.

**Results:**

Colonic segments without fecal transit showed a significant reduction in the tissue content of all mucin subtypes.

**Conclusions:**

All mucin subtypes (neutral, acidic, sulfomucins, and sialomucins) were significantly reduced in colonic segments deprived of fecal stream.

## ARTICLE HIGHLIGHTS

This is the first human study to quantitatively evaluate colonic mucins in diverted bowel.

Diversion colitis was associated with a significant reduction in acidic mucins, neutral mucins, sialomucins, and sulfomucins.

Loss of intestinal transit may compromise the mucosal barrier by reducing protective mucins.

These findings provide new insights into the pathophysiology of diversion colitis and support further investigation of mucin depletion as a potential therapeutic target.

## Summary

**CENTRAL MESSAGE:**

One of the most important colorectal defense mechanisms is the mucus layer, which prevents the vast number of bacteria in the lumen from reaching the sterile layers of the colonic wall. The mucus secreted by the mucosa is composed mainly of mucins, a class of glycoproteins functioning as a defense mechanism by providing both physical and chemical protection against bacteria, toxins, and digestive enzymes. This shields the intestinal wall from inflammation and infection. Certain colonic diseases, such as inflammatory bowel disease, alter the production, composition, and pattern of mucins in the intestinal mucosa.

**PERSPECTIVES:**

A marked reduction in acidic and neutral mucins was observed in patients with diversion colitis. A significant decrease in sialomucins and sulfomucins was also identified in segments of colonic mucosa deprived of intestinal transit. Diversion colitis remains a condition that is frequently underrecognized in colorectal surgical practice, partly because it is often considered a common occurrence in colonic segments deprived of intestinal transit. Consequently, clinical manifestations reported by patients, such as tenesmus, mucorrhea, and hematochezia, are sometimes interpreted as expected or inevitable, which may result in the absence of appropriate therapeutic management.

## VISUAL ABSTRACT

**Figure:**
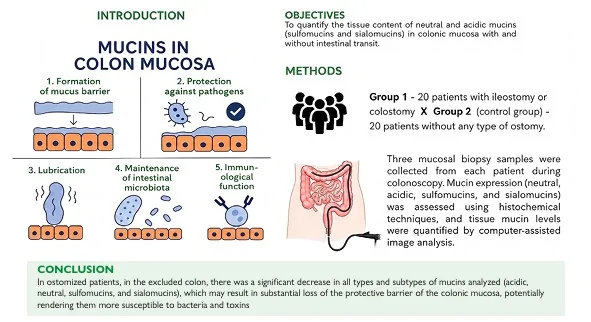

## RESUMO

**Racional:**

Alterações na expressão de mucinas na mucosa intestinal estão entre os achados histológicos mais relevantes no espectro das doenças inflamatórias do cólon. Até o momento, nenhum estudo quantificou a expressão tecidual de mucinas neutras e ácidas, sulfomucinas e sialomucinas em pacientes com colite de exclusão.

**Objetivos:**

Quantificar o conteúdo tecidual de mucinas neutras e ácidas (sulfomucinas e sialomucinas) em mucosa colônica com e sem trânsito intestinal.

**Métodos:**

Foram selecionados 40 pacientes submetidos a colonoscopia eletiva no Hospital de Clínicas – Unicamp. O Grupo 1 incluiu 20 pacientes portadores de ileostomia ou colostomia por mais de 2 meses. O Grupo 2 (controle) consistiu de 20 pacientes sem qualquer tipo de ostomia. Pacientes com doença inflamatória intestinal ou histórico de radioterapia pélvica foram excluídos. Três biópsias de mucosa foram obtidas de cada paciente durante a colonoscopia. A expressão de mucinas neutras e ácidas foi avaliada pelas técnicas histoquímicas de Periodic Acid-Schiff e Alcian Blue, respectivamente. As subcategorias de mucinas ácidas (sulfomucinas e sialomucinas) foram analisadas pelo método Alcian Blue-high iron diamine. A quantificação de todas as subcategorias foi realizada por análise de imagem computadorizada. Testes estatísticos foram aplicados para comparar os níveis teciduais de mucinas entre segmentos com e sem trânsito fecal, adotando-se significância de p<0.05.

**Resultados:**

Observou-se redução significativa no conteúdo tecidual de todas as subcategorias de mucinas nos segmentos colônicos sem trânsito fecal.

**Conclusões:**

Houve diminuição significativa de todas as subcategorias de mucinas (neutras, ácidas, sulfomucinas e sialomucinas) em segmentos colônicos excluídos do trânsito fecal.

## INTRODUCTION

 The inflammatory process that develops in colonic segments excluded from the fecal stream is a consequence of the lack of regular supply of short-chain fatty acids (SCFAs) to the epithelial cells of the colonic mucosa, leading to diversion colitis (DC) or disuse colitis^12^. SCFAs are the main energy substrate utilized by specialized epithelial cells of the intestinal mucosa; therefore, it is believed that the absence of SCFAs due to fecal stream diversion in the colon triggers the colitic process^2,9,13,15^. 

 The epithelium of the large intestine separates the intestinal lumen, harboring a dense bacterial population, from the sterile internal environment, representing one of the most effective functional barriers in living organisms^1,3,17^. This epithelial barrier has a series of cellular structures that provide mechanical protection, as well as immune cells that form a functional immunological barrier. Together, these prevent bacterial agents and other toxins in the intestinal lumen from translocating into the internal milieu, thereby avoiding bacteremia and septicemia^6^. It is now widely accepted that the inflammatory process occurring in the intestinal wall of patients with inflammatory bowel disease (IBD) results from the disruption of this epithelial defense system. 

 One of the most important colorectal defense mechanisms is the mucus layer, which prevents the vast number of bacteria in the lumen from reaching the sterile layers of the colonic wall^10^. The mucus secreted by the mucosa is composed mainly of mucins, a class of glycoproteins that contributes to defense by providing both physical and chemical protection against bacteria, toxins, and digestive enzymes. This shields the intestinal wall from inflammation and infection^5,11,16^. Certain colonic diseases, such as IBD, alter the production, composition, and pattern of mucins in the intestinal mucosa^7,8,13,16^. 

 Mucin production is directly related to adequate energy supply. In colonic segments without fecal transit, that is, without SCFA availability, cellular respiration is altered, leading to the production of large amounts of reactive oxygen species, which cause tissue damage to the mucus layer^11^. This dependency between SCFA-derived energy supply and mucin synthesis has been demonstrated in experimental studies in rats, which confirmed that colorectal segments without fecal stream, and with consequent DC, show reduced levels of neutral mucins, acidic mucins, sulfomucins, and sialomucins, along with alterations in their distribution pattern^13,14,17^. 

 To date, quantitative analysis of neutral mucins, acidic mucins, sialomucins, and sulfomucins in colonic mucosa with and without intestinal transit has not been investigated in humans, making the present study unprecedented. 

## METHODS

### Ethics

 The present study was submitted to and approved by the Ethics Committee of the University of Campinas – Unicamp (CAAE No.: 60265522.0.000.5404). The informed consent form was provided, explained, and signed by both the researcher and the patient. 

### Casuistic

 Forty patients from the Hospital de Clínicas - Unicamp who were already scheduled to undergo elective colonoscopy were selected. All examinations had been previously requested by their attending physicians and were performed independently of this research. Patients were divided into two groups: 

 Group 1 consisted of 20 patients with a previous ileostomy or colostomy for more than two months; 

 Group 2 (control group) included 20 randomly selected patients without previous diversion of the fecal stream. 

 Biopsies were performed solely for research purposes and would not have been collected routinely during colonoscopy. Patients with inflammatory bowel disease or a history of pelvic radiotherapy were excluded from both groups. 

### Histological and histochemical staining

 Fragments collected for histopathological analysis were immersed in 10% neutral formalin buffer (Sigma-Aldrich, St. Louis, MO, USA) for 24 hours, dehydrated by exposure to increasing ethanol concentrations, and embedded in paraffin. Thereafter, sections of tissue were cut at 5 μm on a rotary microtome (Leica Biosystems, Nussloch, Germany), mounted on a glass slide, cleared, hydrated, and stained with hematoxylineosin (HE) to evaluate for the presence of colitis. Analysis of the slides was performed using an optical microscope (Eclipse DS-50, Nikon Inc., Osaka, Japan) at a final magnification of x200. To establish the diagnosis of colitis, as well as the degree of inflammation, the histological slides were analyzed by two blinded observers. 

 The diagnosis of colitis was made based on the presence of five independent histological parameters: reduction in crypt length, number of goblet cells, crypt abscess, intensity of neutrophil infiltration in the mucosa, and epithelial loss. These variables were stratified according to their degree as follows: 0: absent or no alterations; (+): mild intensity; (++): moderate intensity; and (+++): intense. For all variables analyzed, the final value considered for each patient was the mean value after quantification of three distinct histological fields. The inflammatory score for each animal was obtained from the sum of the five variables analyzed. All slides were examined under a light microscope at a final magnification of 200× (Eclipse DS-50, Nikon Inc., Osaka, Japan) by an experienced pathologist specializing in inflammatory bowel disease, who was blinded to the origin of the samples and to the study objectives. The degree of inflammation was graded on a histopathological scale ranging from 0 to 4, according to previously established criteria: 0=no leukocyte infiltration; 1=mild leukocyte infiltration; 2=moderate leukocyte infiltration; 3=marked vascular density and increased thickness of the mucosal and submucosal layers; and 4= transmural leukocyte infiltration, reduced number of goblet cells, high vascular density, presence of ulcerations, and thickening of the mucosal and submucosal layers. 

 The tissue expression of neutral and acid mucins was determined by the histochemical technique of Periodic Acid Schiff (PAS) and Alcian blue (AB), respectively, as previously reported. Neutral mucins were stained magenta, while the acid mucins were stained blue. To identify the subtypes of acid mucins (sulfomucins and sialomucins), the slides were stained using the high iron diamine-Alcian blue (HID-AB) histochemical technique, in accordance with the previous standardized methodology. With the HID-AB technique, sulfomucins were stained brown, and sialomucins were stained blue. 

### Computer-assisted image analysis

 Quantification of tissue acidic mucins was performed using the NIS-Elements image analysis system (Nikon Inc., Japan), version 3.0. The software analyzed color histograms in the red, green, blue (RGB) system to determine the intensity of the selected color, light blue corresponding to the staining of acidic mucins with AB, in pixels per selected field. The final mucin content was expressed as a percentage per field (%/field). The presence of sulfomucins and sialomucins was quantified using the same technique in the same glandular regions. Each measurement was performed in a focal field containing three contiguous and intact glands, and the final value for each sample represented the mean of three histological fields. 

 All statistical analyses were performed using Stata software, version 18.0 (StataCorp LLC, College Station, TX, USA), and a significance level of 5% was adopted for all tests. 

## RESULTS

 Before performing a formal comparison of the variables between the experimental and control groups, data distribution was assessed. The mean values were close to the median values and greater than twice their respective standard deviations for most variables. Furthermore, skewness and kurtosis values were close to the reference values (zero and three, respectively), with the only exception being sialomucins (blue). Finally, the Shapiro-Wilk test yielded non-significant p-values for three out of the four variables analyzed, again except for sialomucins (blue). Therefore, the only variable with an asymmetric distribution was observed in the sialomucin (blue) values, and comparisons involving this variable were performed using nonparametric tests for median comparison (Kruskal-Wallis and sign-rank tests) (Figures 1 and 2, Table 1). 

**Figure 1:**
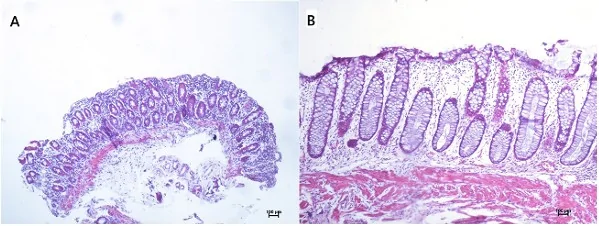
Slides stained using the hematoxylin-eosin technique in group 1 (A) and group 2 (B) demonstrated increased inflammation in group 1 compared with group 2 (the control group). Source: Personal archive.

**Figure 2:**
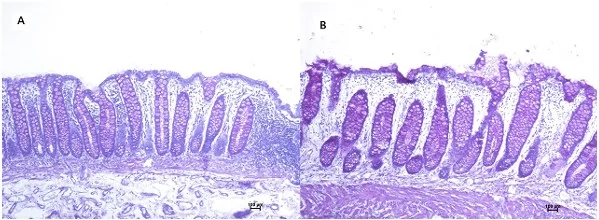
Slides stained using the Periodic Acid-Schiff technique demonstrating a difference in neutral mucins between groups 1(A) and 2(B) (control). Source: Personal archive.

**Table 1:** Evaluation of variable distribution symmetry.

Mucin subtype	Technique	Mean±SD	Median	p-value
Acidic mucins	Alcian Blue	11.7±4.8	12.4	0.628
Neutral mucins	PAS	6.6±2.6	6.9	0.409
Sulfomucins	HID-AB	2.8±2.3	3.4	0.078
Sialomucins	HID-AB	3.8±3.0	2.8	<0.001

SD: standard deviation; PAS: Periodic Acid Schiff; HID-AB: high iron diamine-Alcian blue.

 The mean values of acidic mucins, sulfomucins, and neutral mucins were consistently higher in the control group compared with the experimental group (all p-values=0.01). There was also evidence of a statistically significant difference between the medians of sialomucins in the control and experimental groups, with the control group showing a higher median than the experimental group (p=0.011) (Figure 3). Regarding inflammation, its intensity differed between the control and experimental groups. In the control group, mild inflammation was more prevalent (65%), whereas in the experimental group, moderate inflammation predominated (60%) (Figures 4, 5 and 6, Table 2). 

**Figure 3:**
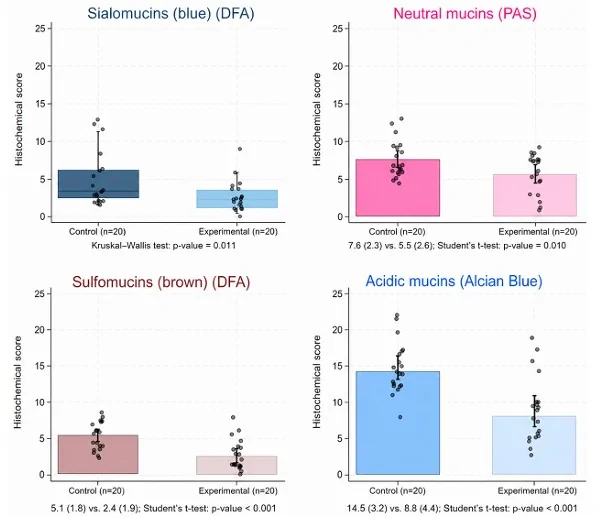
Histochemical quantification of mucins in the colonic mucosa with and without intestinal transit. Box-and-scatter plots comparing acidic mucins (Alcian Blue), neutral mucins (Periodic Acid Schiff), sulfomucins (High iron diamine-Alcian blue), and sialomucins (High iron diamine-Alcian blue) between control subjects (n=20) and patients with diversion colitis (n=20). Each dot represents one patient. Patients with diversion colitis showed significantly lower mucin content than controls in all histochemical analyses. Source: Personal archive. DFA: Direct Fluorescent Antibody; PAS: Periodic Acid Schiff.

**Figure 4:**
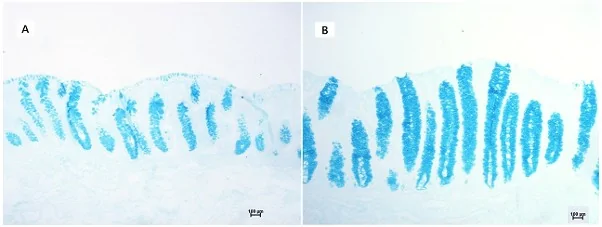
Slides stained using the Alcian Blue technique demonstrating a difference in acidic mucins between groups 1(A) and 2(B) (control). Source: Personal archive.

**Figure 5:**
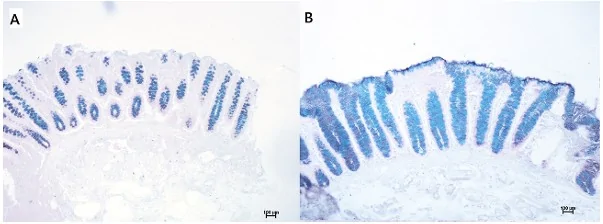
Slides stained using the high iron diamine-Alcian blue/Direct fluorescent antibody histochemical technique, as previously described. Sialomucins appear in blue, demonstrating the difference between group 1 (A) and group 2 (the control group) (B). Source: Personal archive.

**Figure 6:**
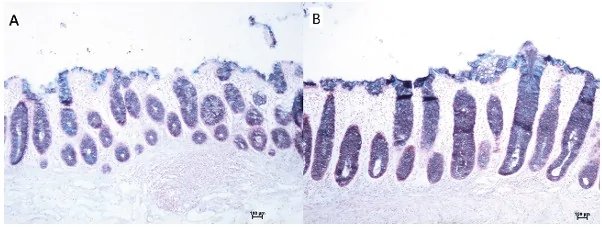
Slides stained using the high iron diamine-Alcian blue/Direct fluorescent antibody histochemical technique, as previously described. Sulfomucins appear in brown, demonstrating the difference between group 1 (A) and group 2 (the control group) (B). Source: Personal archive.

**Table 2:** Comparison of means and medians between the experimental and control groups.

Measurement		Technique	Control group	Experimental group	p-value*
			Mean (SD)	Mean (SD)	
Acidic mucins		Alcian Blue	14.5 (3.2)	8.8 (4.4)	<0.001
Sulfomucins (purple)		HID-AB	5.1 (1.8)	2.5 (1.9)	<0.001
Neutral mucins		PAS	7.6 (2.3)	5.5 (2.6)	0,010
			Median (IQR)	Median (IQR)	p-value†
Sialomucins (blue)		HID-AB	3.4 (2.4–6.1)	2.3 (1.2–3.5)	0.011
			n (%)	n (%)	p-value‡
Inflammation					
	Mild	HE	13 (65.0)	1 (5.0)	<0.001
	Moderate	HE	5 (25.0)	12 (60.0)	
	Severe	HE	2 (10.0)	7 (35.0)	

SD: standard deviation; PAS: Periodic Acid Schiff; HID-AB: high iron diamine-Alcian blue; HE: hematoxylin-eosin; IQR: interquartile range.

*Student’s t-test; †Kruskal-Wallis test; ‡Fisher’s exact test.

## DISCUSSION

 Mucins are high-molecular weight, highly glycosylated glycoproteins that constitute the protective mucus layer covering epithelial surfaces. They play a fundamental role in maintaining the mucosal barrier, contributing to protection against pathogens, modulation of cellular signaling pathways, and regulation of the immune response^10,17^. 

 In the present study, ostomized patients (experimental group) demonstrated a significant reduction in all types of mucins analyzed - acidic and neutral mucins - when compared with the control group. Additionally, a decrease in sialomucins and sulfomucins was observed in patients with diversion colitis compared with healthy individuals (control group with colonic mucosa without histopathological alterations). 

 These findings are consistent with results reported in previous experimental studies conducted in animal models, which also demonstrated decreased mucin expression in colonic mucosa deprived of intestinal transit^1,3,15,17^. 

 Reduced mucins in the excluded colon of ostomized patients may compromise the defensive barrier of the colonic mucosa, rendering it potentially more susceptible to bacterial colonization and the effects of luminal toxins. Another important aspect to consider is the possible relationship between alterations in mucin patterns and the incidence of colorectal cancer. This topic has been investigated for more than three decades^9^, and several studies have demonstrated that qualitative and quantitative alterations in the mucin profile of the colonic mucosa are frequently observed in neoplastic cells. Such alterations may contribute to loss of cellular polarity, activation of signaling pathways associated with tumor growth, increased invasive and metastatic potential, and facilitation of immune evasion mechanisms^4,16^. 

 To the best of our knowledge, this is the first study to perform a comprehensive quantitative analysis of neutral mucins, acidic mucins, sialomucins, and sulfomucins in colonic mucosa with and without intestinal transit. The reduction of these mucins in the excluded colonic segment may weaken the mucosa protective barrier, increasing its vulnerability to bacterial invasion and exposure to luminal toxins. Furthermore, the possibility of an increased risk of colorectal cancer in excluded colonic segments should be considered, given the observed alterations in mucin patterns within these tissues. 

 Considering these findings, further studies aimed at developing therapeutic strategies for diversion colitis are warranted to improve the quality of life of ostomized patients and potentially reduce the incidence of complications following intestinal transit reconstruction procedures. 

 Diversion colitis remains a condition that is frequently underrecognized in colorectal surgical practice, partly because it is often considered a common occurrence in colonic segments deprived of intestinal transit. Consequently, clinical manifestations reported by patients, such as tenesmus, mucorrhea, and hematochezia, are sometimes interpreted as expected or inevitable, which may result in the absence of appropriate therapeutic management. 

## CONCLUSIONS

 A marked reduction in acidic and neutral mucins was observed in patients with diversion colitis. A significant decrease in sialomucins and sulfomucins was also identified in segments of colonic mucosa deprived of intestinal transit. Further studies are required to investigate the consequences of this mucin depletion in the excluded colon. 

## Data Availability

The datasets generated and/or analyzed during the current study are available from the corresponding author upon reasonable request.
